# The Clinical Spectrum, Diagnosis, and Management of GATA2 Deficiency

**DOI:** 10.3390/cancers15051590

**Published:** 2023-03-03

**Authors:** Marta Santiago, Alessandro Liquori, Esperanza Such, Ángel Zúñiga, José Cervera

**Affiliations:** 1 Hematology Department, Hospital La Fe, 46026 Valencia, Spain; santiago_marbal@gva.es (M.S.); such_esp@gva.es (E.S.); cervera_jos@gva.es (J.C.); 2 Hematology Research Group, Instituto de Investigación Sanitaria La Fe, 46026 Valencia, Spain; 3 Centro de Investigación Biomédica en Red de Cáncer (CIBERONC), 28029 Madrid, Spain; 4 Genetics Unit, Hospital La Fe, 46026 Valencia, Spain; zunyiga_ang@gva.es

**Keywords:** GATA2 deficiency, GATA2 haploinsufficiency, germline mutation, predisposition to myeloid neoplasms

## Abstract

**Simple Summary:**

A predisposition to myeloid neoplasms has recently been recognized as a defined clinical entity by the World Health Organization. One of the most well-known syndromes within this group is GATA2 deficiency, which is a highly heterogeneous disorder that can include pulmonary and vascular involvement, immunodeficiency, and myeloid neoplasms. The only curative treatment for this syndrome is allogeneic hematopoietic stem cell transplantation (HSCT), which should be performed in patients with GATA2 deficiency before irreversible organ damage. These patients should be referred to a multidisciplinary team to assess all potential and specific organ-system manifestations that could impact the patient’s treatment, and consultations with appropriate subspecialists should be facilitated. Additionally, genetic testing should be offered to first-degree relatives, particularly those considered for donation when an HSCT with a sibling donor is feasible.

**Abstract:**

Hereditary myeloid malignancy syndromes (HMMSs) are rare but are becoming increasingly significant in clinical practice. One of the most well-known syndromes within this group is GATA2 deficiency. The *GATA2* gene encodes a zinc finger transcription factor essential for normal hematopoiesis. Insufficient expression and function of this gene as a result of germinal mutations underlie distinct clinical presentations, including childhood myelodysplastic syndrome and acute myeloid leukemia, in which the acquisition of additional molecular somatic abnormalities can lead to variable outcomes. The only curative treatment for this syndrome is allogeneic hematopoietic stem cell transplantation, which should be performed before irreversible organ damage happens. In this review, we will examine the structural characteristics of the *GATA2* gene, its physiological and pathological functions, how *GATA2* genetic mutations contribute to myeloid neoplasms, and other potential clinical manifestations. Finally, we will provide an overview of current therapeutic options, including recent transplantation strategies.

## 1. Background

Familial myelodysplastic syndromes (MDSs) and acute myeloid leukemia (AML), also known as hereditary myeloid malignancy syndromes (HMMSs), have been recognized phenotypically for more than a century, with the first molecular basis discovered in 1999 through the identification of germline *RUNX1* mutations [1]. Since then, and recently accelerated by the advent of next-generation sequencing (NGS), a growing number of genes have been associated with germline predisposition to myeloid malignancies, including the *ANKRD26* [2,3,4], *ETV6* [5,6,7], *CEBPA* [8], *DDX41* [9], *GATA2* [10], *RBBP6* [11], *TERT*, *TERC* [12], and, most recently the *SAMD9* [13] and *SAMD9L* genes [14,15]. Although they are traditionally considered very rare entities, it is now known that 4–13% of pediatric and 5–15% of adult MDS/AML cases are caused by germline predisposition [16,17,18,19,20,21]. 

Although most of these entities have only recently been described, the World Health Organization (WHO) incorporated some of them as provisional categories in its fourth revised classification [22]. In recognition of the robustness of data, HMMSs have also been integrated into other guidelines and expert recommendations, such as the Nordic Guidelines and the European Leukemia Network [23,24], highlighting the need to identify, diagnose, and correctly manage patients with hereditary syndromes. Finally, the growing recognition and molecular identification of this subset of myeloid malignancies have led to their being formalized in the most recent revisions by the WHO and the International Consensus Classification (ICC) of myeloid neoplasms [25,26]. The WHO 2022 update reinforces this category and includes it within the group of secondary myeloid neoplasms [25]. On the other hand, the 2022 ICC proposes to place these entities within the category of pediatric and/or germline mutation-associated disorders due to their overlap with other childhood disorders [26]. 

This review focuses on one of these entities, specifically the phenotypic spectrum of patients diagnosed with GATA2 deficiency, recognized as a major myeloid neoplasia predisposition syndrome with pleiotropic manifestations. We discuss the structural characteristics of the *GATA2* gene and describe how its genetic alterations might contribute to the onset of myeloid neoplasms as a result of aberrant induced hematopoiesis [27]. In addition, we will summarize diagnostic clues for proper identification and management of this syndrome.

## 2. *GATA2* Molecular Insights

The *GATA* binding protein 2 (*GATA2*) gene is located on the long arm of human chromosome 3 at cytoband 21.3 (i.e., 3q21.3) and encodes two main isoforms (NM_032638 and NM_001145661) identical in their coding regions, but differing in the 5′ untranslated region [28,29]. The GATA2 protein belongs to the GATA binding factors family, which modulates the expression of several genes by binding to the DNA motif GATA and other transcription factors [30]. This is managed by two highly conserved zinc finger domains (ZF1 and ZF2), which are responsible for the DNA-binding ability of GATA2. In addition, the GATA2 protein contains two transactivation domains, a nuclear localization signal, and a negative regulatory domain [29]. 

The precise role of *GATA2* in hematopoiesis is still not entirely understood. Hematopoietic stem cells (HSCs) found in the bone marrow of *GATA2*^+/−^ mice were found to be impaired in terms of both number and functionality, as evidenced by serial transplantation assays [31]. *GATA2* heterozygosity is associated with decreased proliferation ability and increased quiescence and apoptosis in HSCs [31]. Moreover, *GATA2* haploinsufficiency impairs the function of granulocyte-macrophage progenitors but not that of other committed myeloid progenitors [32]. Despite this, *GATA2*^+/−^ mice do not develop MDS/AML, which makes it challenging to study the impact of *GATA2* haploinsufficiency on leukemic progression in these models. 

On the other hand, the overexpression of *GATA2* results in the self-renewal of myeloid progenitors and hampers lymphoid differentiation in mouse bone marrow [33]. Additionally, the overexpression of *GATA2* promotes proliferation in human embryonic stem cells (hESCs) but quiescence in hESC-derived HSCs [34]. Elevated levels of *GATA2* have been observed in AML patients, both adults and children, who have poor prognoses [35]. These findings indicate that, in addition to its function as a tumor suppressor, *GATA2* may also act as an oncogene when overexpressed.

In line with these data, and focusing on adult hematopoiesis, the GATA2 protein, together with several transcription factors (e.g., FLI1, LMO2, and RUNX1), is involved in HSC survival and self-renewal, thus participating in early lineage commitment. Meanwhile, during hematopoietic differentiation, GATA2 modulates downstream fate decisions by interacting with CEBPA, GATA1, and SPI1 [36,37].

To date, roughly 500 GATA2-deficient patients have been reported, and the syndrome was confirmed to be inherited according to an autosomal dominant pattern in 50% of cases, de novo in 5% of cases, and uncertain in the rest of the cases [38]. This is unexpectedly different from previous studies, in which de novo occurrence was estimated in two thirds of all cases [39,40]. However, there is a lack of a well-characterized series in which segregation studies have been carried out systematically or in which penetrance or expressivity were considered. Therefore, these data should be viewed with caution.

**Figure 1:**
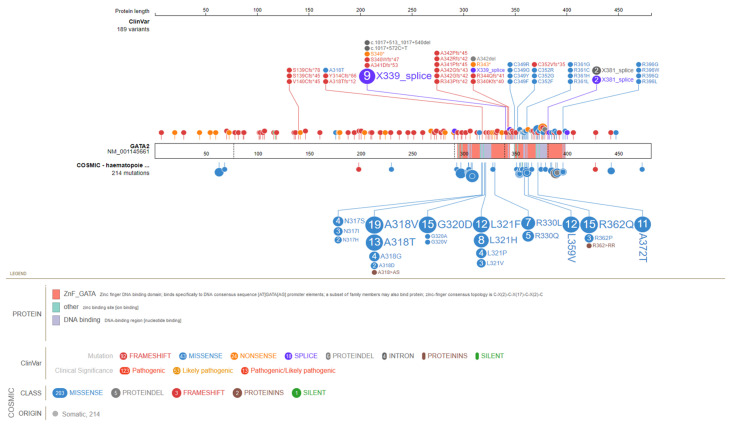
Germline and somatic *GATA2* (likely) pathogenic variants obtained from ClinVar and COSMIC databases, respectively. Somatic variants are restricted to those found in hematopoietic malignancies. Variants were visualized using the ProteinPaint web application (https://pecan.stjude.cloud/home, accessed on 27 January 2023) and colored based on their functional type (e.g., frameshift and missense). Since the effect of splice variants is often undetermined, these were annotated on the position of the closest amino acid that would be involved (e.g., the NM_001145661:c.1018-1G>T variant is annotated as X339_splice). Numbers in circles indicate the number of entries and/or reported cases. All variants are annotated to NM_001145661.

In addition, almost 200 unique (likely) pathogenic variants have been described that can be classified into four groups: truncating mutations (splice site, nonsense, frameshift, and whole-gene deletions) proximal to or within the ZF2 domain; missense mutations within the ZF2 domain; mutations resulting in aberrant mRNA splicing (e.g., synonymous changes) (Figure 1) [38,41,42]; and other regulatory variants, such as those located in the *GATA2* intronic +9.5 kb enhancer site (e.g., c.1017+572C>T, the c.1017+532A>T, and the c.1017+513_1017+540del [c.1017+512del28]), which is essential for hematopoiesis [42,43,44,45,46]. Overall, germline *GATA2* (likely) pathogenic variants are hypothesized to result in haploinsufficiency because truncated alleles lead to clinical phenotypes similar to missense variants [31,45]. Strikingly, some variants have been associated with only partial loss-of-function (p.T354M) or even gain-of-function (p.L359V) mechanisms, suggesting more complex pathways [47,48].

Although most deleterious changes are private, it is possible to recognize some mutational hotspots. Recurrent variants in the extended ZF2 domain have been identified, including p.T354M and p.R396W/Q/W, found in roughly one fifth of the reported cases, as well as the c.1017+572C>T intronic variant, found in 20 patients [38]. 

Germline *GATA2* mutations are usually necessary but not sufficient for myeloid disease development. It has been proposed that different environmental stressors may modify the expression of these germline variants during embryogenesis or after birth, inducing disorder in tissues where limited GATA2 expression is inadequate for their normal cellular function [38]. Particularly in bone marrow (BM), such stressors can lead to certain cytogenetic and molecular alterations that accumulate over time, selecting clonality and triggering myeloid transformation. Indeed, the germline variant can also modify the BM microenvironment, contributing to clonal selection [38].

In patients with progression to a malignant neoplasm, certain cytogenetic and molecular alterations appear recurrently. The most frequent cytogenetic alterations in patients with germline *GATA2*-mutated myeloid neoplasms involve chromosome 7, including its monosomy, partial deletion of 7q and der(1;7)(q10;p10), and trisomy of chromosome 8 [27,40,49]. These neoplasms tend to show fewer somatic mutations and a different molecular landscape compared to non-*GATA2* MDS/AML. The most frequent recurrent somatic mutations identified in *GATA2*-MDS/AML patients are in the *SETBP1, ASXL1,* and *STAG2* genes, and the RAS pathway. By contrast, deleterious *SF3B1, U2AF1, NPM1*, and *FLT3* changes are infrequent in *GATA2*-mutated myeloid neoplasms [21,50,51,52,53,54,55,56,57,58,59,60]. 

Interestingly, *GATA2* can also be mutated in somatic cells of sporadic MDS/AML. Different from germline *GATA2* mutations, which mainly include truncated and ZF2 missense changes, somatic *GATA2* mutations are usually missense variants located in the ZF1 domain (e.g., p.N317-L321 hotspot) or in-frame indels in the C-terminus (Figure 1) [38]. This suggests a likely difference in GATA2 function during the leukemogenic process between germline and somatic cases [61]. Of note, somatic *GATA2* mutations are often associated with both monoallelic and biallelic *CEBPA* somatic mutations [62,63,64]. Additionally, somatic mutations in *GATA2*, although rare, have also been linked to milder forms of the immunodeficiency phenotype observed in patients with germline mutant *GATA2* [65,66].

## 3. *GATA2* Phenotypic Spectrum

Heterozygous pathogenic variants in the *GATA2* gene cause a highly heterogeneous disorder with incomplete penetrance [67]. This may present with immunodeficiency (including monocytopenia with *Mycobacterium avium* complex (MonoMAC) infection and dendritic cell (DC), monocyte, B, and natural killer (NK) lymphoid (DCML) deficiency syndromes); syndromic features, such as congenital deafness and lymphedema (originally defining Emberger syndrome), or pulmonary and vascular involvement [49], and there is a high probability of evolving to MDS and/or AML. In 2011, these diverse clinical syndromes were linked to define a common genetic diagnosis of the GATA2 deficiency syndrome [10,45,68,69].

Except for a few cases, the relationship between genotype and phenotype in these patients is poorly understood due to significant variations in clinical presentation, even among individuals within the same family [41,49]. Therefore, determining the true clinical penetrance of this disorder would require a comprehensive examination of the genotypes of a large number of first-degree relatives of patients. It is worth noting some of the reported phenotype/genotype correlations: (1) patients with noncoding variants (which can account for up to 10% of cases) have been observed to exhibit reduced disease penetrance [41,49,70,71]; (2) the p.T354M variant seemed to be associated with a predominance of myeloid malignancies (83% of cases; 44/53), while p.R398W/Q variants were more commonly associated with immunodeficiency (88% of cases; 23/26) in a relatively large series [38]; (3) there have been indications that complete haploinsufficiency or loss of GATA2 function, rather than missense mutations, may be required for the development of lymphedema [72].

These complex and variable presentations pose a significant challenge for clinicians when diagnosing and managing patients with *GATA2* mutations.

## 4. Hematological Presentation

**Figure 2:**
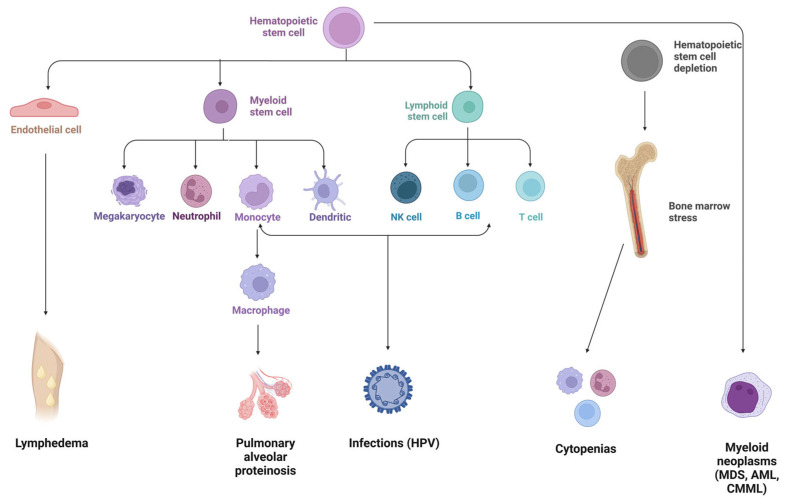
GATA2 deficiency clinical spectrum. HPV, human papilloma virus; MDS, myelodysplastic syndromes; AML, acute myeloid leukemia; CMML, chronic myelomonocytic leukemia. Figure made using https://www.biorender.com/, accessed on 27 January 2023.

The first hematoimmunologic manifestation typically occurs between the second and third decade of life, with a median age that varies in different studies (ranging from 12 to 19 years) [38,39,41,49,71]. While some patients present with cytopenias, immunodeficiency, or BM failure during childhood, others can develop MDS without preexisting clinical features during young adulthood (Figure 2) [27]. 

### 4.1. Bone Marrow Failure

Unlike other germline alterations predisposing to HMMSs that preferentially lead to thrombocytopenia (e.g., *ANKRD26, RUNX1, ETV6*) [73,74,75], neutropenia may be the first and leading form of cytopenia in these patients. Although a decreased white blood cells (WBC) count can lead to a complex differential diagnosis, neutropenia with profound monocytopenia should prompt consideration of GATA2 deficiency [67]. Paradoxically, monocytosis can be the initial presenting sign in patients who develop *GATA2*-related MDS [27]. 

Bone marrow morphology can reveal altered cellularity (hypo- and normal or hypercellular marrow in patients with cytopenia or MDS, respectively), pronounced erythropoiesis, multilineage dysplasia, and fibrosis [40,67]. 

### 4.2. Myeloid Neoplasms

GATA2 haploinsufficiency is a major contributor to MDS/AML in adolescents and young adults. While some patients who develop MDS have a high risk of progressing to AML or chronic myelomonocytic leukemia (CMML), a small subset presents directly with AML [27]. Other reported hematological disorders include acute lymphoblastic leukemia (ALL), juvenile myelomonocytic leukemia (JMML), and myelofibrosis [71,76,77].

The prevalence of GATA2 deficiency is currently unknown, but given the significant disease penetrance and low tolerance to pathogenic mutations in the *GATA2* gene, it is likely that most carriers of the mutation will develop hematologic or immunologic complications over the course of their lifetime. In one study that reviewed 18 published series (>350 individuals), the penetrance of myeloid neoplasms was estimated to reach 75% in *GATA2*-mutated carriers [27], with an increased risk of developing MDS/AML as they aged. The risk of developing MDS/AML was calculated to be 6% at 10 years, 39% at 20 years, and 81% at 40 years in a series of 79 patients [39,71,76]. 

While MDS/AML is the most common neoplasm in GATA2 deficiency, the EWOG-MDS study [49], which included 426 patients, found that *GATA2* germline mutations were present in up to 7% of all pediatric cases with primary MDS and 15% of advanced MDS in examined series [49,78,79]. Monosomy 7 is the most frequent cytogenetic alteration, being present in 37–57% of all patients with *GATA2* MDS and 48–72% of adolescents (>12 years old) with *GATA2* MDS [22,49,76]. Since MDS is very uncommon during childhood, it would seem mandatory to screen all children with this diagnosis for *GATA2* germline mutations [22,49,76]. 

## 5. Immunodeficiency Disorder

GATA2 deficiency is a unique primary immune deficiency that is also known as immunodeficiency 21, DCML, or MonoMAC (OMIM #614172). The immune defect may appear in adult life, as the number of hematopoietic stem and progenitor cells (HSPCs) decreases with age, which makes GATA2 deficiency a unique primary immune deficiency [80]. It is characterized by immunophenotype features resembling those seen in chronic infection or age-related immunosenescence. The spectrum of alterations can include dendritic cell deficiency, monocytopenia, loss of transitional B cells, the absence of CD56 bright NK cells (which presents an altered *CXCL12/CXCR4*-dependent chemotaxis [76,81,82,83,84]), reversed CD4:CD8 ratio, an excess of CD45RA+ CD8+ T cells, and poor-quality humoral response [27,85] despite normal levels of immunoglobulins and an adequate presence of bone marrow plasma cells in most patients [40,86,87]. 

As a result of immune deficiency, GATA2 carriers have an increased frequency of infections, with significant differences in the severity between patients [80]. Due to the deficit and dysfunction of dendritic cells, NK cells, and monocytes/macrophages, the identification of viruses and intracellular pathogens is compromised, leading to the severe spread of viral infections and mycobacterial susceptibility [40,41]. Donadieu and colleagues described severe bacterial infections as the most frequent pathogenic occurrences in *GATA2* carriers, with a cumulative rate of 33% at 20 years and 64% at 40 years [71]. On the other hand, Spinner et al. reported that severe viral infections were the most common ones in their series (70%), in particular those related to the human papilloma virus (HPV), which occurred in about two thirds of carriers [41]. The most important complication derived from underlying HPV infection is the development of recurrent warts or condyloma that can lead to dysplasia and/or squamous carcinoma [88]. Infections with other disseminated pathogens are frequently observed in GATA2-deficient patients, including non-tuberculous mycobacteria, herpes virus (varicella zoster virus, Epstein–Barr virus, and cytomegalovirus), and fungi (invasive aspergillosis, disseminated histoplasmosis, and candidiasis) [41]. 

Therefore, various immunological factors are highly suggestive of GATA2 deficiency and should make the clinician think of this disorder. These include prior immunodeficiency in a patient with MDS, atypical mycobacterial infections in patients with monocytopenia, persistent warts or severe herpes virus infections in cytopenic patients, and loss of B cells and their precursors, especially in patients who develop MDS [27,41,84,85]. 

Eventually, as in other immunodeficiencies, these patients can also present with autoimmune manifestations, described in 11–30% of cases [41,71,89], which may overshadow typical features of GATA2 deficiency and delay the diagnosis. Amarnani et al. reported rheumatological findings in 18% of their GATA2 deficiency cohort, with notable manifestations, including early onset osteoarthritis, piezogenic pedal papules, ankylosing spondylitis, and seronegative erosive rheumatoid arthritis [89].

## 6. Non-Hemato-/Immunologic Manifestations

### 6.1. Pulmonary Involvement

Pulmonary dysfunction is a common finding in up to 50% of patients with GATA2 deficiency [90], even in the absence of hematopoietic disease, leading to progressive compromised pulmonary function with diffusion defects, ventilatory defects, or a mixed pattern, along with significant clinical and radiographic disease [41,71,76,91]. 

In addition to recurrent infections, pulmonary alveolar proteinosis (PAP) is one of the most distinctive lung features. This rare disorder is characterized by the lack of anti-GM-CSF autoantibodies and the accumulation of surfactant proteins and subsequent impaired gas exchange [40]. It results from impaired function of the alveolar macrophages in GATA2-deficiency patients, which are responsible for inadequate clearance, and is associated with increased restrictive ventilatory defects and pulmonary arterial hypertension (PAH) [40,90]. Depending on the studied cohort, PAP and PAH may be present in 4–20% of patients [41,71,90]. 

Therefore, it is recommended to screen patients with PAP and/or immunodeficiency and/or myeloid malignancies without anti-GM-CSF antibodies for *GATA2* alterations. It is important to note that clinical variability within families, including asymptomatic relatives identified through family screening, has also been reported in the case of pulmonary dysfunction [41,90].

Radiographic findings might be unspecific and will depend on the underlying disorder. Several structural abnormalities have been identified on chest computed tomography, including nodular and reticular opacities, ground-glass opacities, consolidations, a “crazy-paving” pattern, subpleural blebbing, and paraseptal emphysema [41,76,90]. 

Although some of the lung manifestations, including PAP, PAH, and underlying infections, can be reversed as a result of an allogeneic hematopoietic stem cell transplantation (allo-HSCT) [41,92,93], it should be noted that HSCT toxicity related to the conditioning regimen and pulmonary graft-versus-host disease (GvHD) can also harm lung function [41,90]. 

Therefore, individuals with GATA2 deficiency should undergo regular, ongoing monitoring of their lung function throughout their lifetime. Although there are no guidelines for the pulmonary follow-up of these patients, it should be individualized and tailored to each patient’s needs. This may involve regular visits to a pulmonologist for symptom monitoring and pulmonary function testing to assess respiratory capacity. Imaging tests, such as chest X-rays or computerized tomography (CT) scans, may also be used to evaluate lung changes. Additionally, if there is suspicion of alveolar proteinosis, a diagnosis confirmation can be made through bronchoscopy with bronchoalveolar lavage (BAL) and/or parenchymal biopsy.

### 6.2. Emberger Syndrome: Dysmorphic Features

Emberger syndrome (OMIM #614038) is characterized by the association of primary lymphedema (a common feature found in 11–20% of *GATA2* carriers, typically affecting one or both lower limbs, frequently involving the genitals in the form of a hydrocele), with AML (often preceded by pancytopenia or MDS), with or without congenital sensorineural hearing loss [38,40,41,68,71,76,94,95,96,97]. 

### 6.3. Other Dysmorphic Features

Additional dysmorphic features that have been described, include hypothyroidism, bilateral syndactyly of the toes, hypotelorism, and epicanthal folds, behavioral disorder, and urogenital malformations, among others [27,41]. 

## 7. Management and Surveillance

### 7.1. Allogeneic-HSCT

Although allo-HSCT is the only curative therapy for the impaired hematopoietic and lymphoid systems of patients with GATA2 deficiency [93,98,99,100], it represents a therapeutic challenge due to disease-associated comorbidities and clinical heterogeneity. Meanwhile, determining who should be candidates for allo-HSCT and when it should be performed (so that the benefits outweigh the risks) are questions that remain under debate [93,100]. Moreover, due to the low prevalence and relatively recent description of GATA2 deficiency syndrome, most outcomes and complications following allo-HSCT have been described in case reports or small series [93,98,100,101,102,103,104]. While some studies have reported an overall survival (OS) rate in 5-year posttransplant patients with clonal events at a rate of 55–60% [41,71,101], other reports have shown superior outcomes after the procedure [98,99,100,103]. Notably, Nichol-Vinueza et al. showed a 4-year posttransplant OS rate of 85.1% [100]. However, these cohorts are not necessarily comparable due to the heterogeneity of conditioning regimens and GvHD prophylaxis, donor type source, HSCT-related risk factors, duration of follow-up, and the clinical status or comorbidities of the GATA2 patient population [98,99,101].

#### 7.1.1. Indications for and Timing of allo-HSCT

While hematologic malignancy development may be the most dangerous complication and a primary indication for transplant, it is not the only one. Restoring normal immunity and lung function is also important in the decision to proceed with SCT [93].

The lack of a genotype-phenotype correlation makes the natural history of GATA2 deficiency unpredictable, to the point that there are patients who become symptomatic after many decades. However, once symptoms appear, survival declines, with a probability of survival by 40 years of 60–80% according to different series [41,71]. In this regard, the ideal time for allo-HSCT should be after the onset of symptoms but before irreversible organ damage occurs [93,98,99], although more specific criteria for the timing need to be defined [105]. While some authors report better outcomes when HSCT is performed earlier after diagnosis and when there are fewer comorbidities [71,100,101], the EWOG-MDS 2017 guidelines on childhood MDS recommend watchful waiting if blood cells are stable and high-risk genetic aberrations are absent [49]. By contrast, other authors go as far as to propose that preemptive allo-HSCT could improve overall outcomes before malignancy develops [106,107,108]. More specific treatment strategies have yet to be fully elucidated. 

There are three major indications for HSCT. Firstly, diagnosis of MDS/AML, however, it is not clear if better timing for HSCT is during the hypocellular MDS phase or when the patients develop cytogenetics abnormalities/excess of blasts [40,41,49,71,98,99,102,103,105]. Secondly, history of severe, recurrent, or treatment-refractory infections, particularly aggressive HPV infection. Relapsed/refractory precancerous or malignant disease due to HPV should be an indication for allo-HSCT. In this sense, considering the iatrogenic immunosuppression after HSCT, rigorous evaluation for HPV must take place before and after transplantation so that surgical and other therapeutic measures can be undertaken in cases with new or persistent disease [93,99,102,103,104,105]. Thirdly, progressive lung injury from infection and PAP, which leads to deteriorated lung function [93,99,102,103,105].

#### 7.1.2. Conditioning, Graft Source, and Donors

Transplanting GATA2-deficient patients is a controversial topic due to the variable disease progression and the timing of HSCT [100,109]. Although nonmyeloablative HSCT can reverse clinical manifestations and was the strategy used in the earlier years, relapse rates, engraftment failure, and late graft rejections led to the consideration of more intensive conditioning regimens [93,102]. In this regard, several reports have demonstrated similar outcomes when using myeloablative regimens in patients with mutated and wild-type (wt) GATA2 [49,98,101]. However, in patients with low-stage and hypocellular MDS, myeloablation may not be necessary due to low rates of relapse [98], and the intensity can be reduced by using a controlled approach [110]. Therefore, some authors propose that the choice of conditioning scheme choice for GATA2-deficient patients should be based on the patient’s MDS phenotype and cytogenetics [101,103,105,110]. 

The donor source constitutes a critical variable in the outcome of HSCT. Although it is still unclear which donor source will yield better outcomes for GATA2-deficient patients [102], it has been suggested that bone marrow should be preferred over peripheral blood, while umbilical cord blood should be avoided [102,103]. Matched related donors remain the best choice, although haploidentical HSCT could be an appropriate alternative [103,110].

#### 7.1.3. HSCT-Derived Complications

Bortnick et al. conducted a study of 65 cases and found that pediatric patients with GATA2 deficiency had a similar risk of transplant-related toxicity (TRT) or transplant-related mortality (TRM) as compared to those with wt *GATA2* [98]. However, they also reported that three patients developed transplant-associated microangiopathy, which might indicate a distinct endothelial vulnerability in GATA2 patients, consistent with the known role of *GATA2* in the perturbation of normal vascular development [41,111,112]. Simonis et al. conducted a systematic review of 183 patients (median age 23 years) from January 2010 until March 2018 and reported that the risk of TRT was not higher in patients with GATA2 deficiency compared to those without it [93]. Similarly, Hofmann et al. reported no differences in TRM and overall organ toxicity between a pediatric cohort with GATA2 deficiency and controls [101]. However, they did observe a small number of serious and unusual infectious/immunologic complications and neurologic toxicities in the GATA2 population, as well as a higher rate of thrombotic events in GATA2 patients, with complete resolution after transplantation [101]. 

Although information about GvHD is often not available in these series, it seems that the proportion of patients with acute or chronic GvHD is similar to that of other transplant cohorts [93,100,101]. Reducing the severity of both acute and chronic GvHD is being evaluated in GATA2 deficiency patients with promising outcomes by administering post-cyclophosphamide (PTCy) after HSCT, as seen in wt GATA2 patients [100,103,105]. However, when HSCT is indicated but no preexisting malignancies are present, strategies to prevent GvHD are of the greatest importance, as there is no advantage to this complication [110]. 

In summary, considering that there are no formal recommendations on the indications for allo-HSCT, conditioning regimen, GvHD prophylaxis, donor source, and antibiotic prophylaxis in GATA2-deficiency patients, the decision to perform an allo-HSCT requires careful and individualized management [99]. Although treatment-related morbidity is manageable in these patients, an individualized approach should be taken into consideration for optimal outcomes.

### 7.2. Antibiotic Prophylaxis, Immunoglobulins, and Vaccination

Prior to performing allo-HSCT, it is crucial to effectively treat any severe infections to create a favorable environment for the transplanted donor stem cells to thrive [105]. Although opportunistic infections that manifest before transplantation do not seem to pose a major issue in terms of overall outcomes, patients are typically kept on antibiotic prophylaxis to prevent such infections. While most case reports of allo-HSCT do not provide details on the antibiotic prophylaxis regimen, in a study by Simonis et al., patients treated for non-tuberculous mycobacterium before HSCT took prophylactic azithromycin until the time of transplant and for about one year afterward [93]. For patients still receiving treatment for active infection at the time of HSCT, antimycobacterial drugs were administered for 6–12 months after the transplant [93]. Spinner et al. also recommend azithromycin for all patients with GATA2 deficiency even before HSCT is indicated [41].

Although rare, GATA2 patients may experience humoral deficiency [87,113]. In such cases, immunoglobulin replacement may be necessary [40].

Due to the high susceptibility of patients to HPV and the potential for recurrent and life-threatening oncogenic HPV lesions, early vaccination is likely to be beneficial [40,41,71].

### 7.3. Surveillance

Given the complexity of information available on GATA2 deficiency syndrome and other HMMSs, patients should be referred to multidisciplinary teams that include physicians who are well-versed in these conditions. This would facilitate the assessment of potential organ-system manifestations that could impact the patient’s treatment, and promote consultation with appropriate subspecialists. 

Since most patients with symptomatic GATA2 deficiency will eventually require an allo-HSCT, close monitoring is crucial in order to perform the procedure before organ damage occurs [93,98]. Therefore, a donor search should be conducted as soon as the deficiency is diagnosed, with systematic testing of potential relatives considered for donation [71,99]. 

Allo-HSCT can eradicate clonal malignancy, restore normal hematopoiesis, clear underlying infections, and improve pulmonary symptoms and function in patients with PAP [93,98,103]. However, it cannot reverse extra-hematopoietic manifestations of GATA2 deficiency, so patients remain at risk for non-hematopoietic issues and will require lifelong follow-up [101,105]. It is worth mentioning that HPV can persist after allo-HSCT, so gynecologists play an important role in guiding the management and surveillance of these patients [104,114], especially during the period of immunosuppression following the procedure. 

### 7.4. Family Monitoring

Genetic testing should be offered to first-degree relatives, particularly to potential donors of HSC progenitors, to identify asymptomatic carriers with GATA2 deficiency. According to some authors, hematological surveillance of carriers should include annual bone marrow analysis with morphological, cytogenetic, and molecular evaluation to prevent the appearance of new driver acquisitions [99]. Moreover, some researchers recommend avoiding exposure to corticosteroids and immunosuppressive drugs and monitoring pulmonary function regularly to prevent complications [27].

### 7.5. Genetic Counseling

It is important to note that genetic counseling should be offered to family members who test positive for *GATA2* mutations to help them understand the implications of the diagnosis and the potential risks of passing the condition on to their own children, and they should receive proper information about the different reproductive or prenatal diagnostic options.

## 8. Conclusions

Recognizing GATA2 deficiency in clinical care is crucial for several reasons [115]. Firstly, an accurate diagnosis can help patients understand their specific disorder and avoid inappropriate treatments. Secondly, a genetic diagnosis can aid in selecting the most suitable HSCs donor for an allo-HSCT. TShirdly, identifying GATA2 syndrome can impact treatment recommendations and disease management for patients and their families. As patients with this condition face various complications affecting many systems, HSCT is often an attractive therapeutic option. The choice of therapy largely depends on the patient´s age, the availability of a compatible donor, and any co-existing medical conditions. Thus, early and accurate diagnosis of these patients allows for tailored therapy.

## 9. Future Directions

As awareness of GATA2 deficiency grows within the scientific community, early diagnosis will help in avoiding unnecessary diagnostic procedures and enable tailored strategies, for both treatment and surveillance [49]. 

Moreover, we may be able to identify patients who are at high risk of transforming to myeloid malignancies based on factors such as molecular alterations, cytogenetic evolution, or severity of cytopenias. By managing these patients early, we can aim for better outcomes before organ dysfunction occurs. 

## Data Availability

Not applicable.
